# AIMD-driven insights into the thermodynamic stability and thermoelectric performance of Li_2_YCuX_6_ (X = Cl, Br, I) double perovskites

**DOI:** 10.1039/d6ra05396c

**Published:** 2026-07-31

**Authors:** Malik Muhammad Asif Iqbal, Muhammad Kaleem, Asif Nawaz Khan, Muhammad Abaid Ullah

**Affiliations:** a Department of Chemistry, University of Okara 56300 Pakistan mmasif101@gmail.com; b Department of Physics, International Islamic University H-10 Islamabad 44000 Pakistan kaleemphy@gmail.com; c Material Modeling and Simulation Lab, Department of Physics, University of Science & Technology Bannu 28100 Khyber Pakhtunkhwa Pakistan; d Department of Physics, University of Okara 56300 Pakistan

## Abstract

The accelerating global demand for low-carbon and renewable energy is driving the search for efficient materials for photocatalytic water splitting. Here, density functional theory (DFT) calculations using the CASTEP code are employed to systematically investigate the structural, electronic, optical, and mechanical properties of Li_2_YCuX_6_ (X = Cl, Br, I) double perovskites. Structural stability is confirmed by negative formation energies (−1.37, −1.29, and −1.20 eV per atom) and Goldschmidt tolerance factors within the favourable range of 0.772–0.786. Dynamical stability is further validated through phonon dispersion analysis, which shows the absence of imaginary frequencies across the Brillouin zone, confirming lattice stability under ambient conditions. All compounds exhibit thermal stability at 300 K without significant structural distortion, as confirmed by *ab initio* molecular dynamics (AIMD) simulations. Electronic structure analysis reveals semiconducting behaviour, with GGA-PBE band gaps of 1.47 eV for Li_2_YCuCl_6_, 1.50 eV for Li_2_YCuBr_6_, and 1.57 eV for Li_2_YCuI_6_. Optical calculations indicate strong absorption in the UV-visible range with Li_2_YCuI_6_ exhibiting a maximum absorption coefficient of 1.9 × 10^5^ cm^−1^ at 9.7 eV. The calculated band-edge positions suggest favorable energetic alignment for photocatalytic water-splitting reactions. However, the present results should be regarded as a first-principles screening assessment, and further investigations are required to fully establish photocatalytic performance. These findings provide theoretical guidance for future experimental and theoretical investigations.

## Abstract 2

The accelerating global demand for low-carbon and renewable energy is driving the search for efficient materials for photocatalytic water splitting.

## Graphical abstract

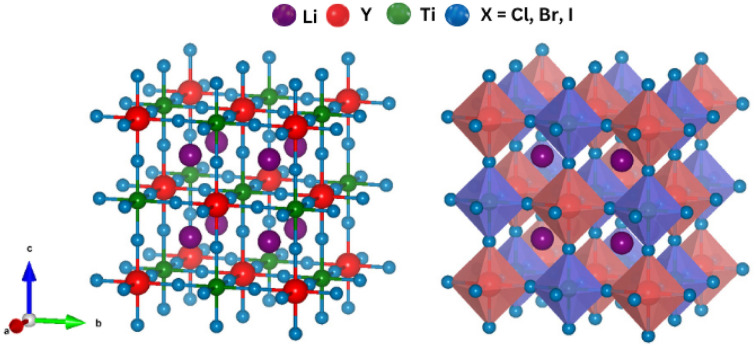

## Introduction

Solar-driven water splitting has gained significant attention, with halide perovskite materials emerging as promising candidates due to their favorable optoelectronic properties. These materials exhibit three key characteristics that make them particularly promising: strong light absorption, tunable band gaps, and excellent capacity for charge transport. Their potential to efficiently convert solar energy into chemical fuels positions them as strong candidates for sustainable hydrogen generation *via* water splitting.^1^ Recently, double perovskite compounds have attracted significant attention because of their adaptable structural frameworks and tunable electronic characteristics, enabling their use in diverse advanced technological applications. Double perovskites have shown considerable potential in fields such as optoelectronics, photovoltaics, spintronics, magneto-optic devices, and magnetoresistance materials, driven by their tunable band structures, diverse magnetic ordering, and inherent chemical flexibility.^2^ Double perovskites have attracted growing attention owing to their excellent structural, mechanical, and thermal stability. Moreover, their optoelectronic properties can be precisely tailored through compositional engineering, enabling band gap modulation suitable for photovoltaic and other advanced optoelectronic applications. Beyond these merits, double perovskites exhibit pronounced optical responses, including efficient light absorption, reflection, refraction, and photoconductivity across a broad range of the electromagnetic radiation ranges.^3^ These attributes have motivated extensive research into their synthesis and functional integration across diverse platforms, including photocatalysis and light-harvesting technologies. Since the initial discovery of the perovskite structure by Gustav Rose in 1839,^4^ significant progress has been made in tailoring their chemistry and physical behaviour for emerging energy and electronic applications.

This study presents a comprehensive investigation of the structural, mechanical, electronic, and optical properties of Li_2_YCuX_6_ (X = Cl, Br, I) double perovskites, to evaluate their potential as light-harvesting materials specifically to split water through photocatalysis. The stability of proposed compounds in the perovskite phase was confirmed through elastic constants and phonon dispersion analyses, indicating mechanical and thermodynamic robustness. Detailed electronic structure calculations reveal band gap variations and transition characteristics as a function of halide composition, providing critical insights into their suitability for visible-light absorption. Frequency-dependent dielectric functions were used to derive key optical properties, including photo absorption coefficient and reflectivity spectra. Additionally, excitonic properties such as binding energies and radii were quantified to assess charge separation efficiency, which is an essential factor in photocatalytic performance. This integrated approach offers valuable predictive findings regarding the design of lead-free perovskite materials tailored for sustainable solar-to-hydrogen energy conversion.

### Computational technique

DFT calculations were carried out using the CASTEP code within the plane-wave pseudopotential framework^5^ to investigate the structural, electronic, optical, mechanical, thermodynamic, and photocatalytic properties of Li_2_YCuX_6_ (X = Cl, Br, I) double perovskites. A plane-wave cutoff energy of 340 eV was employed for wavefunction expansion, and ultrasoft pseudopotentials were adopted to describe core-valence interactions. Exchange–correlation effects were treated using the Perdew–Burke–Ernzerhof (PBE) functional within the generalized gradient approximation (GGA). The GGA-PBE functional was selected because it has been extensively employed in the investigation of halide perovskites and double perovskites, providing a reliable balance between computational efficiency and predictive accuracy for structural, electronic, optical, and thermodynamic properties. Although the PBE function generally underestimates the absolute values of band gaps, it effectively captures the electronic structure trends, chemical bonding characteristics, and relative band-edge positions that are essential for comparative studies of related perovskite materials. Considering the closed-shell electronic configurations of Li^+^, Y^3+^, Cu^+^, and halide ions, the investigated compounds are expected to exhibit non-magnetic behavior. Therefore, non-spin-polarized calculations were adopted throughout this study. Brillouin-zone sampling was performed using a 6 × 6 × 6 Monkhorst–Pack *k*-point mesh, and structural optimizations were carried out with the Broyden–Fletcher–Goldfarb–Shanno (BFGS) minimization scheme.^6^ Convergence thresholds were set at 1 × 10^−5^ eV per atom for total energy, 0.03 eV Å^−1^ for maximum force, and 1 × 10^−3^ Å for maximum atomic displacement. All compounds crystallize in a cubic double perovskite structure of the A_2_BB′X_6_ with space group *Fm*3̄*m* (no. 225) and lattice angles *α* = *β* = *γ* = 90°. The conventional unit cell consists of 10 atoms, comprising Li, Y, Cu, and halogen (Cl, Br, I) ions. The electronic configurations considered were Li: [He] 2s^2^, Y: [Kr] 4s^2^ 3d^1^, Cu: [Ar] 4s^2^ 3d^9^, Cl [Ne] 3s^2^ 3p^5^, Br [Ar] 4s^2^ 4p^5^ and I: [Kr] 5s^2^ 5p^5^. Coordination numbers for Li, Y, Cu, and X were taken as 12, 6, 6, and 6, respectively. The corresponding ionic radii used for structural modeling were Li^1+^ (1.06 Å), Y^3+^ (0.90 Å), Cu^1+^ (0.77 Å), Cl^1−^ (1.81 Å), Br^1−^ (1.96 Å), and I^1−^ (2.20 Å).^7^ This optimized structural framework served as the basis for subsequent evaluations of stability, optoelectronic performance, and photocatalytic activity.^8^ The thermal stability of the system was assessed using *ab initio* molecular dynamics (AIMD) simulations with the pw.x module of Quantum ESPRESSO. The study of ionic mobility involved the use of Verlet integration. The investigation into dynamic stability was carried out using Phonopy software in conjunction with Quantum ESPRESSO. Molecular dynamics and phonon dispersion simulations were performed using the primitive unit cell and a dense *k*-mesh of 8 × 8 × 8. The SCF accuracy was set to 10^−8^ Ry (AIMD) and 10^−9^ Ry (phonons), and the energy and force convergence thresholds were set to 10^−4^ Ry and 10^−3^ bohr per a.u., respectively. To ensure computational accuracy, plane-wave cut-off energies of 80 Ry (AIMD) and 85 Ry (phonons) were employed.

## Results and discussion

### Structural characteristics

**Fig. 1:**
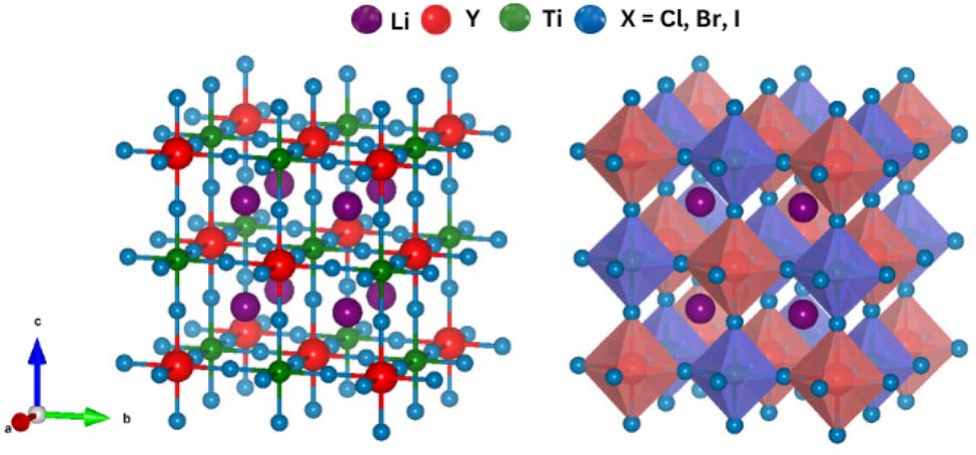
Optimized Li_2_YCuX_6_ (X = Cl, Br, I) structure in cubical phase double perovskites.

The double perovskite framework exhibits inherent structural flexibility, enabling it to adopt diverse geometries such as cubic, monoclinic, tetragonal, and hexagonal phases. In this work, the symmetry and lattice features of Li_2_YCuX_6_ (X= Cl, Br, I) were analyzed through unit cell geometry optimization. Fig. 1 illustrates the optimized crystalline arrangement of the compounds. All Li_2_YCuX_6_ compositions were found to crystallize in the cubic *Fm*3̄*m* (225) space group.^9^ The atomic positions within the unit cell were identified as follows: Li at 
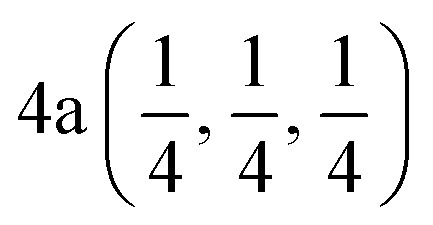
, Y at 4b (0, 0, 0), Cu at 
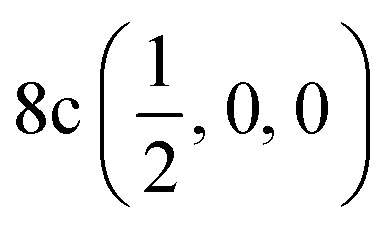
, and X (Cl, Br, I) at 24e 24e (0.255606,0,0). Lattice parameters play a pivotal role in determining the electronic band gap, as the relative displacements between positively charged cations and negatively charged anions are strongly governed by interatomic spacing.^10^ The optimized lattice constants were calculated to be 10.33 Å, 10.91 Å, and 11.75 Å for Li_2_YCuCl_6_, Li_2_YCuBr_6_, and Li_2_YCuI_6_, respectively. This systematic increase reflects the progressive substitution of Cl^−^ by heavier halogens, consistent with the larger ionic radii of Br^−^ and I^−^ compared to Cl^−^. As no theoretical or experimental data are currently available for the lattice parameters and electronic characteristics of these compounds, the present DFT results provide first-principles predictions of their structural properties. The calculated ground-state parameters, including lattice constants, bond angles, unit cell volumes, and densities, are summarized in Table 1.

**Table 1:** The computed structural parameters for Li_2_YCuX_6_ double perovskites

Compounds	Lattice constant *a* = *b* = *c* (Å)	Volume (Å^3^)	*ρ* (g cm^−3^)	*τ*_G_	*µ*	Δ*H*_f_	*E*_g_ (eV)
Li_2_YCuCl_6_	10.33	1101.94	2.28	0.786	0.492	−1.37	1.47
Li_2_YCuBr_6_	10.91	1300.33	3.29	0.780	0.454	−1.29	1.50
Li_2_YCuI_6_	11.75	1624.51	3.79	0.772	0.405	−1.20	1.57

While initial geometry optimization confirms the stability of Li_2_YCuX_6_ double perovskites, a more rigorous evaluation is required to establish their suitability for technological applications. Structural stability plays a decisive role in determining applicability for optoelectronics, photocatalysis, and solar energy conversion. For both perovskites, such as ABX_3_ and A_2_BB′X_6_ types, stability and formability are commonly assessed using Goldschmidt tolerance factor (*τ*_G_)^11^ and octahedral factor (*µ*).^12^ In this work, the crystal structures of Li_2_YCuX_6_ were systematically analyzed through these geometric parameters, with *τ*_G_ and *µ* values computed using eqn (1) and (2), respectively.^13^1
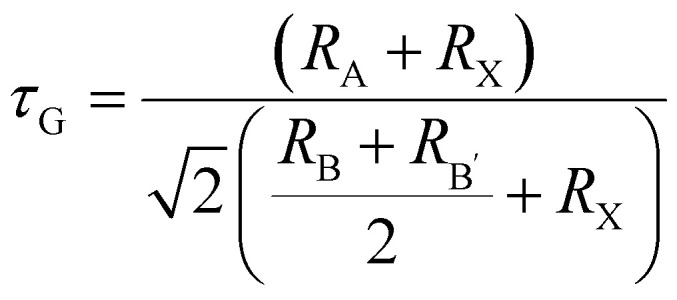
2
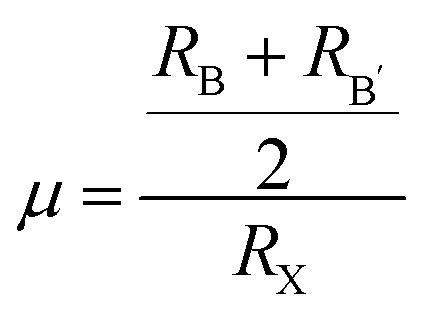
here *R*_A_, *R*_B_, *R*_B′_, and *R*_X_ represent the Shannon ionic radii of Li, Y, Cu, and Cl, Br, I, respectively. These radii were used to evaluate the geometric parameters, with *R* representing the ionic size of each constituent element in the double perovskite lattice. The structural stability of double perovskite compounds is assessed by *τ*_G_ and *µ*, which are considered optimal within the ranges of 0.71–1.00 and 0.41–0.75, respectively.^14,15^ For Li_2_YCuCl_6_, Li_2_YCuBr_6_, and Li_2_YCuI_6_ the calculated *τ*_G_ values are 0.786, 0.780, and 0.772, while the corresponding *µ* values are 0.492, 0.454, and 0.405. These values lie within or close to the established stability ranges, thereby confirming the structural feasibility of these compounds in the double perovskite phase, as summarized in Table 1. Although the *µ* value of Li_2_YCuI_6_ lies slightly below the ideal threshold, it remained acceptable given the distortion tolerance characteristic of halide perovskites. Overall, the computed geometric parameters validate the structural stability of Li_2_YCuX_6,_ underscoring their potential for robust and efficient optoelectronic applications.

Formation energy (Δ*H*_f_) provides a key thermodynamic descriptor for evaluating the thermodynamic stabilization of a compound, as it reflects the energy released or absorbed during its formation. A negative Δ*H*_f _ signifies that double perovskite is energetically favorable and thermodynamically stable. Formation energies of Li_2_YCuX_6_ double perovskites were determined using eqn (3).^16^3
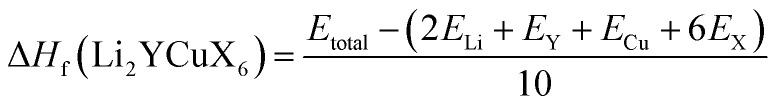
In this expression, *E*_total_, *E*_Li_, *E*_Y_, *E*_Cu_, and *E*_X_ correspond to the total energy of the compound and reference energies of Li, Y, Cu, and halogen atoms (Cl, Br, I), respectively. The calculated Δ*H*_f _ are −1.37, −1.29, −1.20 eV per atom for Li_2_YCuCl_6_, Li_2_YCuBr_6_, and Li_2_YCuI_6_, respectively. As presented in Table 1, all three compounds display negative formation enthalpies Δ*H*_f_, thereby confirming their thermodynamic stability. These results indicate that the studied double perovskites are energetically favorable and potentially synthesizable.

### Electronic properties

Electronic properties, particularly the energy band gap (*E*_g_) and density of states (DOS), are central to understanding material behavior at the atomic scale. A detailed analysis of Li_2_YCuX_6_ was undertaken, as insights into these properties are essential for predicting performance and optimizing functionality in advanced technologies.^17^ The *E*_g_ governs photon absorption, electrical conductivity, optical response, and defect tolerance. It should be noted that the electronic properties reported in this work were obtained using the GGA-PBE exchange–correlation functional. Although GGA-PBE is widely employed for the investigation of halide perovskites and provides reliable trends in electronic structure and band-edge characteristics, it is known to underestimate absolute band-gap values. Therefore, the calculated band gaps should be interpreted primarily as comparative descriptors for screening and understanding the relative electronic behavior of the Li_2_YCuX_6_ series. More advanced approaches, such as hybrid-functional (HSE06) or many-body GW calculations, may provide improved quantitative accuracy and constitute an important direction for future studies. In the present study, the Fermi level (*E*_F_) is marked by a dashed line, and the electronic band structures were computed along the high-symmetry path (X–R–M–Γ–R) in the first Brillouin zone.^18^

**Fig. 2:**
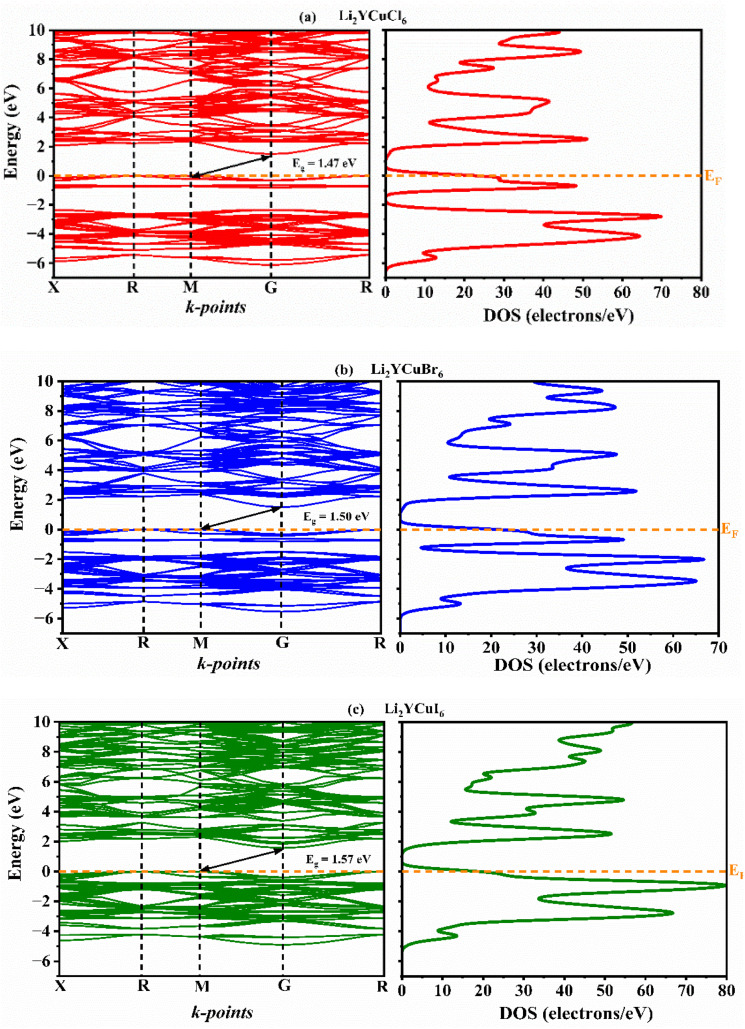
Electronic band structure of Li_2_YCuX_6_ (X = Cl, Br, I).

Li_2_YCuCl_6_, Li_2_YCuBr_6_, and Li_2_YCuI_6_ exhibit indirect band gaps of 1.47, 1.50, and 1.57 eV, respectively, as shown in Fig. 2a–c, confirming their semiconducting character. The variation in *E*_g_ originates from substitution at the X-site: increasing the halogen ionic radius from Cl^−^ to I^−^ alters the electronic environment and bonding, thereby modulating the band gap. These values represent the energy separation between the valence-band maximum (VBM) and the conduction-band minimum (CBM). The relatively flat dispersion near the VBM and CBM suggest favorable electronic characteristics that may influence charge-carrier transport behaviour. However, explicit evaluation of carrier mobility and electron–hole recombination dynamics would require additional calculations beyond the scope of the present study. The absence of electronic states around the Fermi level is consistent with the semiconducting nature of the investigated compounds and indicates a well-defined separation between the valence and conduction bands. Collectively, these results demonstrate that Li_2_YCuX_6_ compounds combine structural and electronic features well suited for optoelectronic and solar-energy applications.

To further elucidate the electronic structure of Li_2_YCuX_6_, the total density of states (TDOS) was evaluated, as shown in Fig. 2. The *E*_F_ is aligned at 0 eV and indicated by a vertical dashed line, with states below and above corresponding to VB and CB, respectively. All compounds display a vanishing TDOS at *E*_F_, confirming their semiconducting nature.^19^ The dominant TDOS contributions in the VB region extend from approximately −5 eV to 0 eV. Among the series, Li_2_YCuI_6_ exhibits the most pronounced peak intensity near the VB maximum, reflecting enhanced hybridization relative to the Cl^−^ and Br^−^based analogues. A sharp increase in TDOS just above *E*_F_ corresponds to the onset of CB states, while the clear separation between the VB maximum and CB minimum further corroborates the indirect band gaps observed in the band-structure calculations. These results confirm that halogen substitution at X-site modulates the electronic density distribution and directly influences the band-gap characteristics.

**Fig. 3:**
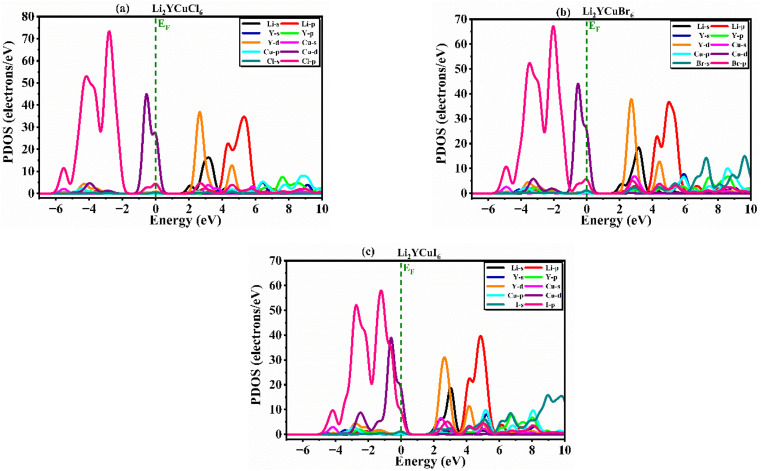
(a–c) PDOS of Li_2_YCuX_6_ (X = Cl, Br, I) double halide perovskite.

The calculated partial density of states (PDOS) for Li_2_YCuX_6_ (X = Cl, Br, I) is shown in Fig. 3a–c. The VB region near the *E*_F_ is dominated by halogen p-states, highlighting their decisive role in shaping the electronic properties of these compounds. The Cu 3d orbitals contribute strongly from approximately −5 to 2 eV, spanning both the VB and CB, highlighting their central involvement in optical transitions and charge transport. The presence of sharp peaks and pronounced d-state intensities indicates partial d–p hybridization between Cu and the halogen atoms, which enhances the covalent bonding character. Specifically, the halogen p-orbitals hybridize with Cu d-states and Y s/p-states, giving rise to sp^2^- and sp^3^-type hybridization, particularly evident within the VB region.

Among the halides, Li_2_YCuCl_6_ exhibits more localized electronic states, whereas Li_2_YCuBr_6_ and Li_2_YCuI_6_ display broader features, reflecting progressive orbital delocalization and stronger p–d overlap down the halogen group. The enhanced delocalization observed in Li_2_YCuI_6_ points to increased orbital mixing and hybridization, which is expected to improve carrier mobility and photon absorption. These orbital interactions and hybridizations provide crucial insight into the bonding framework, electronic transitions, and optoelectronic response of Li_2_YCuX_6_. In the calculations, valence electrons from Li (2s^1^), Y (4d^1^, 5s^2^), Cu (3d^10^, 4s^1^), and halogens Cl (3s^2^, 3p^5^), Br (4s^2^, 4p^5^), and I (5s^2^, 5p^5^) were explicitly considered. Collectively, these features suggest that the Li_2_YCuX_6_ double perovskites emerging as potential materials for next-generation photovoltaic and optoelectronic applications, driven by strong d–p hybridization and favorable band alignment. While the present electronic-structure analysis provides valuable insight into the band topology, orbital hybridization, and semiconducting nature of Li_2_YCuX_6_, practical photocatalytic performance is also influenced by defect states, carrier lifetimes, charge-transport characteristics, and surface reaction kinetics. These factors were not explicitly investigated in the current work and therefore warrant further study to establish a more comprehensive understanding of the photocatalytic behavior of these materials.

### Dynamical stability

**Fig. 4:**
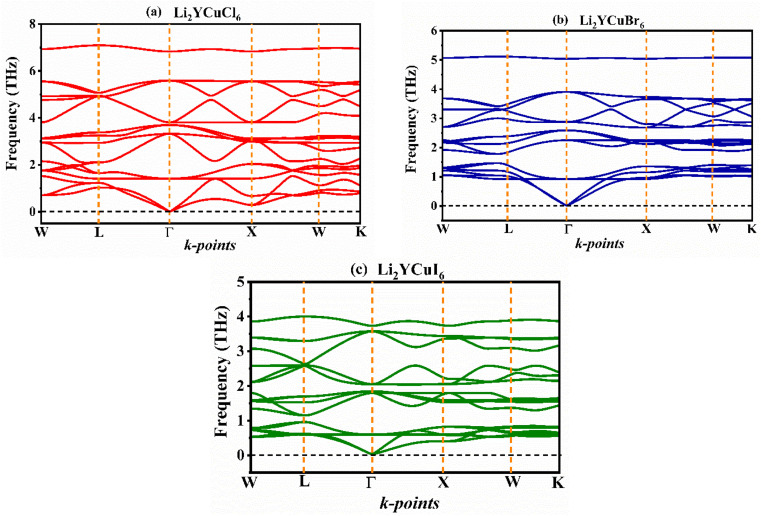
Phonon dispersions describing (a) Li_2_YCuCl_6,_ (b) Li_2_YCuBr_6,_ and (c) Li_2_YCuI_6_ double perovskites.

Phonon dispersion analysis provides a fundamental approach for evaluating the dynamical stability of crystalline materials and probing their vibrational characteristics, which directly influence thermal conductivity, mechanical integrity, and long-term structural reliability under operational conditions. In the context of photocatalytic water splitting, dynamical stability ensures that the crystal lattice remains robust under light irradiation and thermal fluctuations, thereby maintaining the integrity of the photocatalyst and sustaining its efficiency during repeated redox reactions.^20^ To evaluate this aspect, the phonon dispersion relations of Li_2_YCuX_6_ (X = Cl, Br, I) were computed along high-symmetric directions in the Brillouin zone (Fig. 4a–c). Notably, none of the compounds exhibit imaginary frequencies (negative modes) across the Brillouin zone, thereby confirming their dynamical stability at ambient pressure and temperature.^21^ Among the three materials, Li_2_YCuCl_6_ exhibits the highest maximum phonon frequency (∼7.9 THz), followed by Li_2_YCuBr_6_ (∼5.1 THz) and Li_2_YCuI_6_ (∼4.6 THz). This systematic decrease reflects the increasing atomic mass of the halogen, which induces softening of vibrational modes due to weaker bond stiffness and lower force constants. Such trends are consistent with the well-established mass effect on lattice dynamics, where heavier atoms suppress vibrational frequencies. In addition, the phonon spectra reveal relatively flat optical branches across all compounds. The flatness signifies reduced phonon group velocities; a feature commonly associated with low lattice thermal conductivity. This behavior is advantageous for energy applications, as reduced thermal conductivity minimizes heat loss and mitigates localized thermal stresses during exothermic or endothermic hydrogen storage processes.

A distinct separation between acoustic and optical branches is especially pronounced in Li_2_YCuBr_6_, suggesting different vibrational responses between the heavier halogen sublattice and the lighter cation framework. Such phonon gaps can strongly influence phonon scattering, anharmonic interactions, and ultimately the thermal transport behavior of the lattice factors that are essential for optimizing hydrogen storage efficiency.^21^ In summary, the phonon dispersion results confirm that Li_2_YCuX_6_ (X = Cl, Br, I) double perovskites are dynamically stable across the halogen series. The combined features of stable acoustic modes, systematic softening with heavier halogens, low phonon group velocities, and mode separation underscore their thermally robust and low-conductivity lattices. These attributes are highly desirable for water splitting applications, where both structural resilience and controlled thermal properties are essential for sustained performance.

### *Ab initio* molecular dynamics (AIMD) calculations

**Fig. 5:**
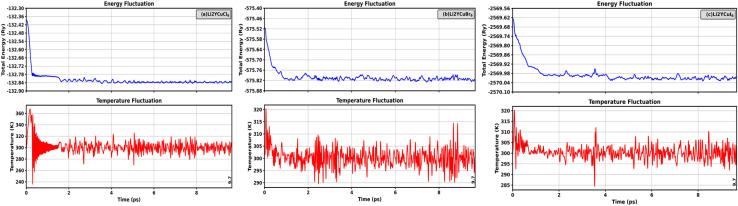
AIMD total-energy traces *versus* time for (a) Li_2_YCuCl_6_, (b) Li_2_YCuBr_6,_ and (c) Li_2_YCuI_6_ perovskite hydrides.

AIMD simulations were employed to check the stability of the compounds that were examined at room temperature. A temperature of 300 K was selected because it represents ambient operating conditions that are most relevant for the practical utilization of photocatalytic and optoelectronic materials. The primary objective of the AIMD analysis was to evaluate the structural integrity and thermodynamic stability of Li_2_YCuX_6_ (X = Cl, Br, I) under realistic working conditions. Although simulations over a wider temperature range could provide additional insight into high-temperature behavior and thermal robustness, such an investigation is beyond the scope of the present study and is proposed for future work. This approach yielded a more precise and comprehensive evaluation. Fig. 5(a–c) show how the potential energy changes at each stage of the simulation. The figures provide estimated AIMD simulation results for the halide double perovskite Li_2_YCuX_6_ (X = Cl, Br, I) at 300 K. The calculations comprise 10 000 steps (9.7 ps), with each step taking 0.96 fs. The energy fluctuations remain almost consistent over the simulation time, suggesting that there is no discernible structural deformation or energy drift. This confirms that all perovskites exhibit excellent thermal stability at room temperature, aligning well with earlier studies^22–24^ and suggesting strong potential for practical applications under ambient conditions.

### Elastic and mechanical characteristics

The mechanical robustness of a photocatalyst is a key factor in evaluating its suitability for water splitting applications. In this study, the mechanical behavior of Li_2_YCuX_6_ (X = Cl, Br, I) was analyzed, focusing on elasticity, stiffness, ductility, and anisotropy. An ideal material must maintain its structural integrity and withstand mechanical failure under prolonged exposure to light, repeated thermal cycling, and chemically reactive environments. Elastic constants serve as fundamental parameters that provide direct insight into the intrinsic mechanical stability of crystalline solids. In cubic systems, the independent elastic constants include *C*_11_, *C*_12_, and *C*_44_, which were computed using eqn (4), and the resulting values are summarized in Table 2. Physically, *C*_11_ quantifies the resistance to uniaxial deformation along the principal crystallographic axes, *C*_12_ characterizes the lateral or transverse response under longitudinal stress, and *C*_44_ describes the shear resistance of the lattice, closely linked with hardness and rigidity.^25^ To ensure mechanical stability, the computed elastic constants were evaluated using the Born stability criteria for cubic crystals, which require that:

4 *C*_11_ − *C*_12_ > 0, *C*_11_ + 2*C*_12_ > 0, *C*_11_ > 0, *C*_44_ > 0, *C*_12_ < *B* < *C*_11_

as expressed in eqn (4). Satisfaction of these conditions ensures that the structure resists spontaneous mechanical distortion under stress.^26^ The results confirm that all three Li_2_YCuX_6_ (X = Cl, Br, I) compounds fulfill these criteria, highlighting their mechanical robustness and potential to withstand operational stresses in photocatalytic environments.

**Table 2:** Elastic constants (*C*_*ij*_) and Born stability criterion for Li_2_YCuX_6_

Compounds	Elastic constants (*C*_*ij*_)			Born stability criteria			Stability
	*C*_11_	*C*_12_	*C*_44_	*C*_11_ > 0	*C*_44_ > 0	*C*_11_+ 2*C*_12_ > 0	
Li_2_YCuCl_6_	42.82	24.16	7.61	42.82	7.61	91.14	Stable
Li_2_YCuBr_6_	33.58	21.36	6.78	33.58	6.78	76.3	Stable
Li_2_YCuI_6_	29.73	14.89	6.83	29.73	6.83	59.51	Stable

Table 2 confirms that all Li_2_YCuCl_6_ (X = Cl, Br, I) compositions satisfy the Born stability criteria, thereby validating their mechanical stability under ambient pressure. Among them, Li_2_YCuCl_6_ exhibits the highest elastic constants (*C*_11_ = 42.82 GPa, *C*_44_ = 7.61 GPa), indicative of a strong atomic bonding framework and pronounced rigidity. Such robustness is advantageous for withstanding mechanical stresses during repeated photoexcitation cycles in photocatalytic reactors. A systematic decrease in elastic stiffness is observed with increasing halogen size from Cl to I, which can be attributed to Cu–X bond elongation and consequent lattice softening. While this reduces resistance to deformation, it may facilitate enhanced photon-induced lattice dynamics and charge carrier mobility. Importantly, all three compounds retain positive shear constants 
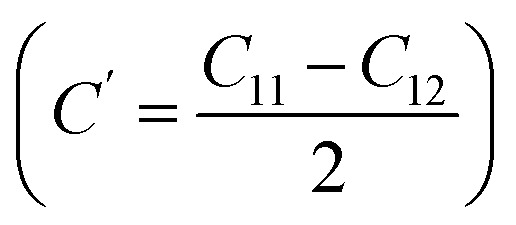
, confirming their thermodynamic stability (*C*′ > 0) and suitability for photocatalytic water-splitting applications.^27^

Bulk modulus (*B*), shear modulus (*G*), and Young's modulus (*E*) were evaluated using the Voigt–Reuss–Hill approximation (eqn (5)–(7)).^28^5
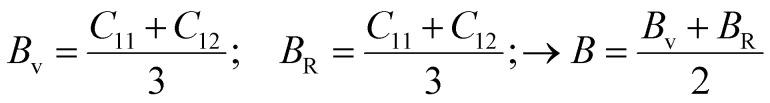
6
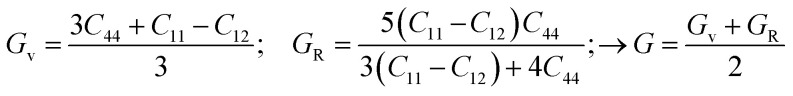
7
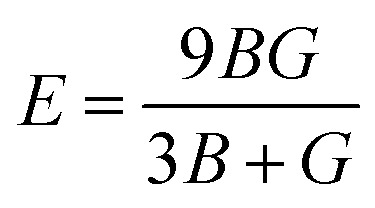


The bulk modulus (*B*) reflects a material's resistance to uniform compression and indicates its volumetric stability during photocatalytic reactions involving gas evolution (H_2_, O_2_). Among the designed compounds, Li_2_YCuCl_6_ demonstrates the highest *B* value (30.38 GPa), highlighting its superior resistance to structural collapse under internal pressure. The shear modulus (*G*) and Young's modulus (*E*) provide insight into deformation resistance and overall stiffness. High stiffness is essential for maintaining surface geometry under photon pressure and charge migration, thereby preventing microcracks that could impair catalytic performance.^29^ Consistent with this, Li_2_YCuCl_6_ also exhibits the highest *G* (8.26 GPa) and *E* (22.71 GPa), underscoring its excellent mechanical robustness as mentioned in Table 3. In comparison, Li_2_YCuI_6_ is mechanically softer; however, its *G* and *E* values remain above 7 GPa and 18 GPa, respectively, which are sufficient to withstand operational stresses in photocatalytic environments.

**Table 3:** Elastic constants calculated for Li_2_YCuX_6_

Compounds	*B* (GPa)	*G* (GPa)	*E* (GPa)	*B*/*G*	*ν*	*A* ^U^	*C*_p_
Li_2_YCuCl_6_	30.38	8.26	22.71	3.68	0.37	0.05	16.55
Li_2_YCuBr_6_	25.43	6.51	17.98	3.91	0.38	0.01	14.58
Li_2_YCuI_6_	19.84	7.06	18.93	2.81	0.34	0.008	8.06

Ductility is a crucial property for ensuring that a photocatalyst can withstand thermal expansion, vibrational stresses, and mechanical agitation without fracturing. Pugh's ratio (*B*/*G*) serves as a reliable criterion for evaluating this property, where compounds with *B*/*G* > 1.75 are classified as ductile and fracture-resistant, an essential requirement for repeated use in aqueous catalytic environments.^26^ All three Li_2_YCuX_6_ (X = Cl, Br, I) compounds exhibit high ductility, with *B*/*G* values well above the threshold: 3.68 (Cl), 3.91 (Br), and 2.81 (I). This pronounced ductility enhances their ability to resist crack propagation, particularly under cyclic illumination where photoinduced lattice vibrations are significant. Notably, Li_2_YCuBr_6_ demonstrates the highest ductility, suggesting its strong potential for long-term durability in practical water-splitting applications.

Cauchy pressure (*C*_p_ = *C*_11_ − *C*_12_), another established indicator of ductility, is positive for all Li_2_YCuX_6_ (X = Cl, Br, I) compositions.^30^ This confirms their ability to undergo plastic deformation without fracture, a property critical for sustaining catalytic activity during mechanical handling or repeated gas evolution cycles. Poisson's ratio 
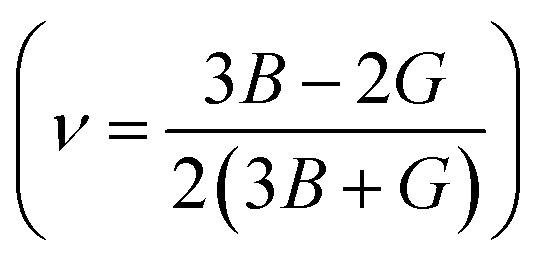
 gives further insights into the bonding characteristics and deformation behavior of materials under tensile stress.^31^ As presented in Table 3, all compounds exhibit *ν* values in the range of 0.34–0.38, consistent with ionic solids. Such an ionic bonding character is particularly beneficial for photocatalytic performance, as it strengthens the dielectric response and facilitates efficient charge carrier separation. Moreover, the intermediate *ν* values indicate that these materials can dissipate mechanical strain effectively, minimizing localized stress accumulation a particularly valuable trait for photocatalysts and nanostructured systems.

Photocatalytic materials in heterogeneous reactor environments are frequently subjected to multidirectional stresses, making an isotropic mechanical response highly desirable. The Universal Elastic Anisotropy Index 
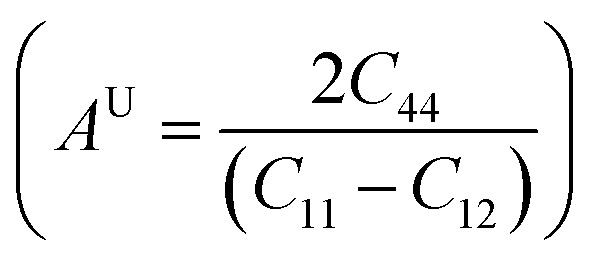
 quantifies this property, where *A*^U^ = 1 indicates perfect isotropy and deviations reflect anisotropy.^30^ All Li_2_YCuX_6_ compounds exhibit low anisotropy, with Li_2_YCuI_6_ showing an almost isotropic behavior (*A*^U^ = 0.008), while Li_2_YCuCl_6_ and Li_2_YCuBr_6_ also remain within minimal anisotropy limits (0.05 and 0.01, respectively). Such mechanical uniformity ensures reliable performance across different crystallographic orientations, enhancing the stability of thin films and layered photocatalyst architectures under fluid flow and vibrational loads. It should be noted that the elastic and mechanical properties reported in this work correspond to ambient pressure and temperature conditions. In general, the application of external pressure strengthens interatomic interactions and reduces lattice compressibility, leading to an increase in the elastic constants (*C*_11_, *C*_12_, and *C*_44_), bulk modulus, shear modulus, and Young's modulus. Conversely, increasing temperature enhances lattice vibrations and thermal expansion, which typically weaken interatomic bonding and reduce the elastic stiffness of crystalline materials. Consequently, Li_2_YCuX_6_ (X = Cl, Br, I) compounds are expected to exhibit greater mechanical rigidity under compression and a gradual softening at elevated temperatures. Although a detailed pressure- and temperature-dependent investigation is beyond the scope of the present study, the mechanically stable nature of these compounds at ambient conditions suggests that they may retain favorable mechanical performance over a reasonable range of operating conditions. Future studies employing quasi-harmonic approximation or finite-temperature first-principles calculations would provide further insight into their thermomechanical behavior.

### Optical responses

The optical response of a photocatalyst dictates its ability to absorb solar photons, generate electron–hole pairs, and subsequently drive the redox reactions required for splitting water into H_2_ and O_2_. In this study, the optical properties of Li_2_YCuX_6_ (X = Cl, Br, I) were investigated using DFT within the GGA-PBE framework. The key parameters, including the frequency-dependent complex dielectric function, absorption coefficient, reflectivity, extinction coefficient, refractive index, and optical conductivity, were evaluated to determine their suitability for visible-light photocatalysis.

**Fig. 6:**
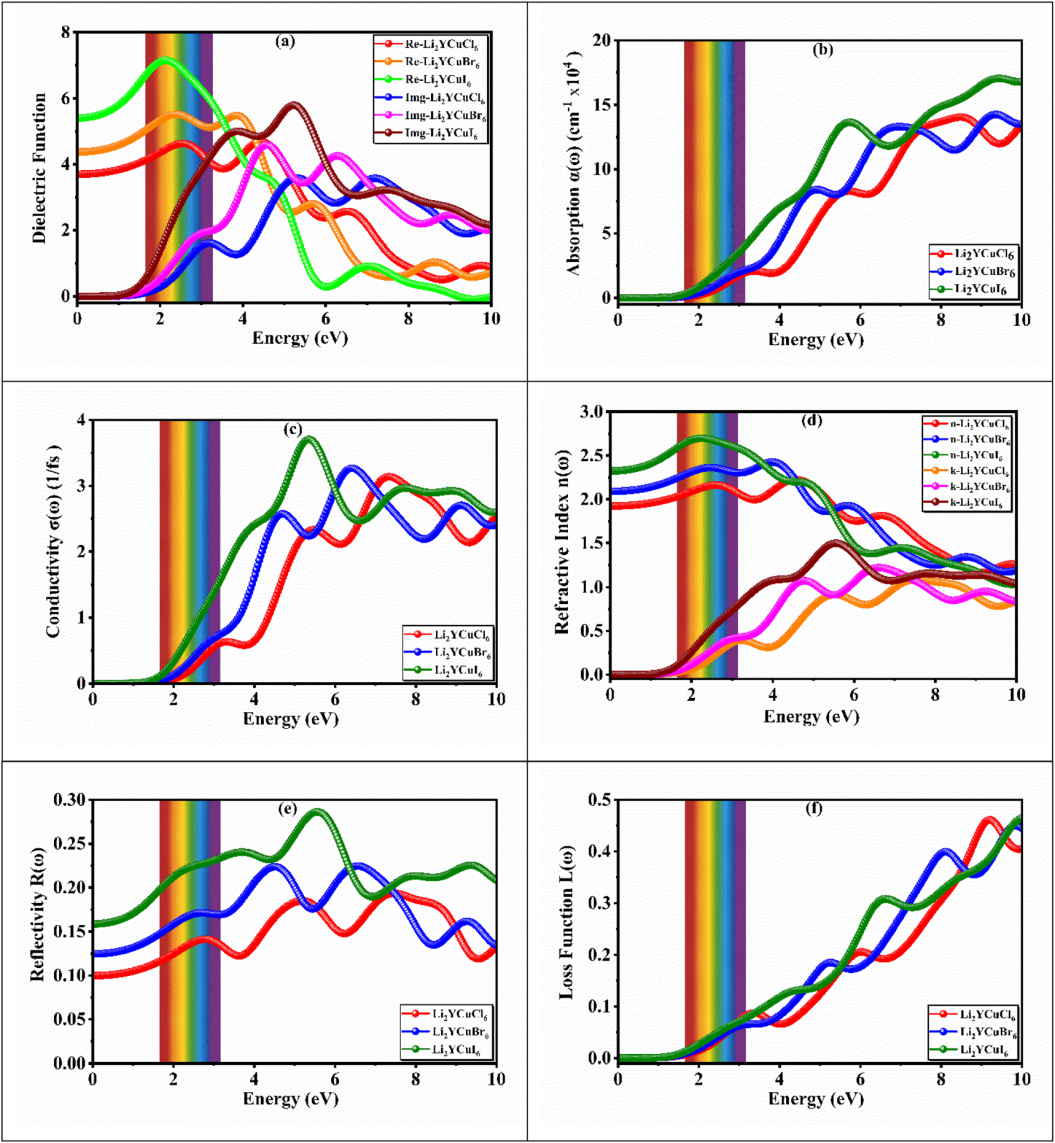
Optical properties of Li_2_YCuX_6_ (X = Cl, Br, I) (a) dielectric function (b) absorption (c) conductivity (d) refractive index (e) reflectivity (f) energy loss function.

The dielectric function, *ε*(*ω*) = *ε*_1_(*ω*) + *iε*_2_(*ω*), provides fundamental insight into light-matter interactions. The real part *ε*_1_(*ω*), corresponds to dispersion and polarization, while the imaginary part *ε*_2_(*ω*), accounts for absorption *via* interband electronic transitions as illustrated in Fig. 6a.^32^ The static dielectric constants, *ε*_1_(0), exhibit moderate values across all halides, suggesting reasonable dielectric screening. A higher *ε*_1_(0) generally suppresses electron–hole recombination, thereby improving charge utilization during photocatalysis. The *ε*_2_(*ω*) spectra display pronounced peaks in the ultraviolet and near-visible regions for Li_2_YCuCl_6_, Li_2_YCuBr_6_, and Li_2_YCuI_6_, which originate mainly from Cu–X and Y–X hybridized state transitions. It is worth noting that the optical response of Li_2_YCuX_6_ is primarily governed by electronic transitions involving Cu-, Y-, and halogen-derived states located near the valence- and conduction-band edges. In contrast, Li-derived states contribute minimally in the vicinity of the Fermi level and therefore have a limited direct influence on the fundamental optical transitions. Consequently, moderate variations in Li positions resulting from thermal fluctuations are not expected to significantly alter the overall absorption characteristics or dielectric response of the material. Nevertheless, substantial Li redistribution at elevated temperatures may locally modify the charge-density distribution and polarization behavior, which could lead to minor changes in optical properties. Such temperature-dependent Li-ion dynamics and their influence on the optical response constitute an interesting subject for future investigation. Importantly, the absorption onset of *ε*_2_(*ω*) coincides with the calculated band gaps, confirming the indirect nature of optical transitions and validating their potential for visible-light-driven activity.

The absorption coefficient
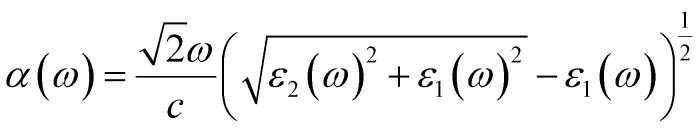
, reflects a material's ability to harvest sunlight and generate charge carriers.^33^ In photocatalytic systems, high absorption coefficient values in the visible-light region is critical for efficient solar energy utilization. For Li_2_YCuCl_6_ and Li_2_YCuBr_6_, the absorption edges appear near 1.9–2.2 eV, whereas Li_2_YCuI_6_ exhibits a red-shifted onset around 1.7 eV as shown in Fig. 6b, extending further into the visible spectrum. This shift originates from its narrower band gap and the enhanced polarizability of iodine atoms. While all compositions display strong ultraviolet absorption due to high-energy electronic transitions,^34^ Li_2_YCuI_6_ stands out with markedly higher absorption intensity in the visible region, identifying it as the most promising candidate for solar-driven photocatalytic water splitting. These results highlight that visible-light absorption in Li_2_YCuX_6_ can be systematically tuned through halogen substitution, offering a strategic pathway for optimizing solar energy harvesting.

The real part of the optical conductivity
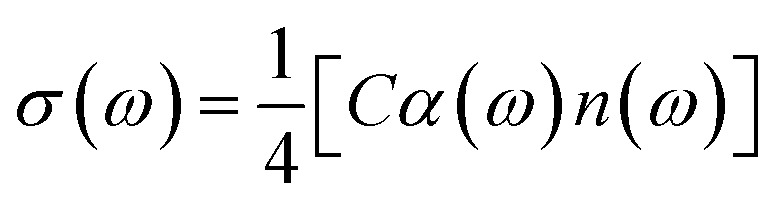
, quantifies the rate of electronic excitation and the probability of interband transitions under incident light.^35^ A higher *σ*(*ω*) in the visible region reflects stronger photon–electron interactions, thereby facilitating electron excitation from VB to the CB. Among the studied compounds, Li_2_YCuI_6_ exhibits the highest optical conductivity within the visible spectrum, consistent with its narrower bandgap and elevated *ε*_2_(*ω*) response as shown in Fig. 6c. This enhanced optical activity implies more efficient generation of photocarriers under visible irradiation, a critical requirement for sustaining hydrogen evolution (HER) and oxygen evolution (OER) reactions during photocatalytic water splitting.

The refractive index
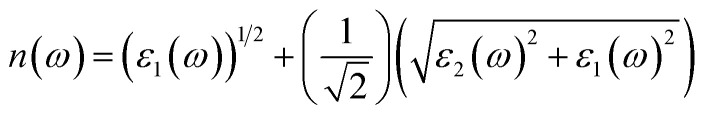
, describes how light propagates through the medium.^34^ A high refractive index at low photon energies (near 0 eV) indicates strong light matter interaction, which is advantageous for photon trapping and extended optical path lengths in photocatalytic thin films.^36^ The static refractive indices, *n*(0), are moderately high for all three compounds, following the order Li_2_YCuI_6_ > Li_2_YCuBr_6_ > Li_2_YCuI_6_, consistent with the increasing polarizability of the halogen atoms. This progression enhances photon confinement and can contribute to higher photocatalytic quantum efficiency. As photon energy increases, *n*(*ω*) decreases gradually and approaches unity at higher energies, indicating a typical optical dispersion behavior as shown in Fig. 6e.^37^ These optical characteristics confirm that Li_2_YCuX_6_ compounds possess the ability to channel and concentrate incident light effectively in the active region, thereby supporting efficient photon absorption and subsequent photocatalytic activity.

Low reflectivity is a desirable trait in photocatalysts, as it minimizes photon loss through surface reflection. The reflectivity spectra
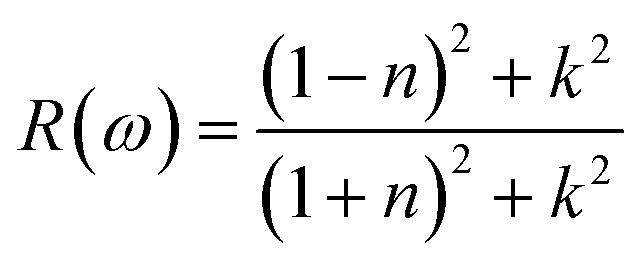
,^38^ for Li_2_YCuX_6_ compounds remain relatively low (≈30%) in the visible region, with slightly higher peaks in the ultraviolet. This indicates that a considerable fraction of incident solar radiation is absorbed rather than reflected, thereby enhancing photocatalytic efficiency. The extinction coefficient, which represents the imaginary part of the complex refractive index and governs the attenuation of light intensity within the material, is given as: 
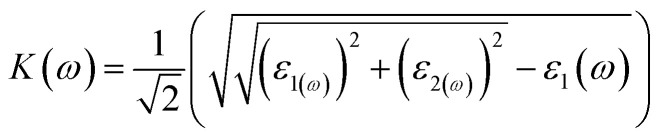
^38^ Its variation closely mirrors that of *ε*_2_(*ω*), with strong peaks in the UV region and moderate absorption tails extending into the visible spectrum, as shown in Fig. 6f. Notably, Li_2_YCuI_6_ exhibits the highest *k* values in the 1.5–3.0 eV range, confirming its superior ability to harness visible-light photons. This characteristic makes it the most promising candidate among the series for solar-driven water splitting applications.

### Photocatalytic properties

Water splitting driven by solar energy represents a clean and efficient route for producing hydrogen, directly transforming sunlight into chemical energy in the form of H_2_ and O_2_. In this process, semiconductor-based photocatalysts play a central role by absorbing incident photons and initiating redox reactions required for water splitting.^39^ For efficient photocatalysis, the material must exhibit strong visible-light absorption, efficient generation and separation of electron–hole pairs, effective charge transport, and chemically active sites for water dissociation. Materials with suitable electronic band structures, wide optical response, and stable surface properties are therefore ideal candidates. The reaction proceeds through two half-reactions.^8^

8 2H^+^ + e^−^ → H_2_ (HER)

9 2H_2_O → O_2_ + 4H^+^ + 4e^−^ (OER)

10 2H_2_O → 2H_2_ + O_2_ (overall water splitting)

HER efficiency is influenced by several factors, including band-gap energy, spectral absorption range, charge-carrier transport, recombination dynamics, surface reaction kinetics, and redox potential alignment. In the present work, the photocatalytic potential is evaluated primarily from bulk electronic structure, optical properties, and band-edge alignment. For optimal hydrogen generation, the material must possess a sufficiently narrow band gap to harness visible light, exhibit long carrier lifetimes with suppressed electron–hole recombination, and maintain conduction and valence band positions favorably aligned with the HER and OER redox levels.

Specifically, hydrogen production through photocatalysis is critically dependent on the band edge alignment of the catalyst. For spontaneous water splitting to occur, the CBM must lie at a more negative potential than the H^+^/H_2_ reduction level (0 V *vs.* NHE, pH = 0), enabling photogenerated electrons to reduce protons.^5^ Simultaneously, the VBM should lie at a more positive potential than the H_2_O/O_2_ oxidation level (1.23 V *vs.* NHE), ensuring sufficient driving force for water oxidation.^40^ To assess the photocatalytic suitability of Li_2_YCuCl_6,_ Li_2_YCuBr_6_, and Li_2_YCuI_6_, the band edge positions were calculated using the following empirical relations (eqn (11)–(13)).11
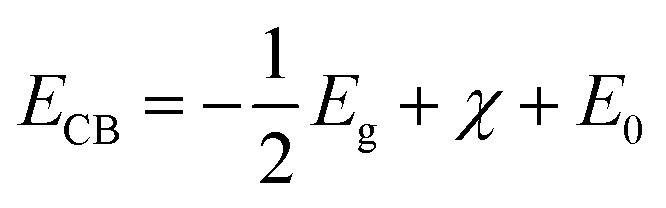

12 *E*_VB_ = *E*_g_ + *E*_CB_

**Fig. 7:**
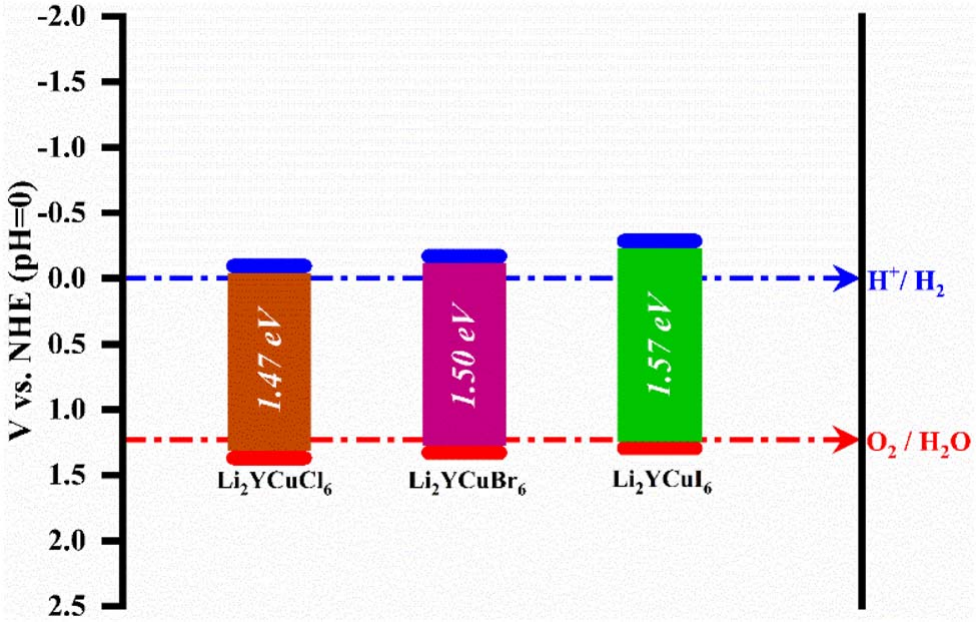
Schematic for evaluation of band edge potentials of Li_2_YCuCl_6_ and Li_2_YCuBr_6_, and Li_2_YCuI_6_.

13
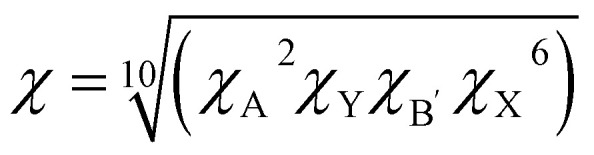
where *χ* represents the electronegativity of the compound, *E*_0_ is the reference vacuum potential of the normal hydrogen electrode (−4.5 eV), and *E*_g_ represents the bandgap energy value.^40^ The computed electronegativity values are 2.8921, 2.8726, and 2.8471 for Li_2_YCuCl_6_, Li_2_YCuBr_6_, and Li_2_YCuI_6_, respectively. Employing these values, the band edge potentials (CBM and VBM) for each compound were calculated. All three materials exhibit CBM values located at negative potentials, favorable for proton reduction to H_2_. The calculated CBM potentials are: −0.095 eV for Li_2_YCuCl_6_, −0.17 eV for Li_2_YCuBr_6_, and −0.285 eV for Li_2_YCuI_6,_ as shown in Fig. 7. Since efficient hydrogen evolution requires CBM to be positioned below the H^+^/H_2_ reduction potential (0 V *vs.* NHE, pH = 0), all three materials satisfy this criterion. Notably, Li_2_YCuI_6_ possesses the most negative CBM among the investigated compounds, suggesting a stronger thermodynamic driving force for proton reduction. However, the actual HER activity also depends on surface catalytic properties, charge-carrier dynamics, and reaction kinetics, which were not explicitly investigated in the present work.

Furthermore, the calculated VBMs of 1.37 eV (Li_2_YCuCl_6_), 1.33 eV (Li_2_YCuBr_6_), and 1.295 eV (Li_2_YCuI_6_) all exceed the H_2_O/O_2_ oxidation potential (1.23 V *vs.* NHE). This confirms their suitability for the OER, with Li_2_YCuCl_6_ showing the highest oxidation driving force. In addition to favorable band edge alignments, these compounds possess narrow band gaps that enable efficient absorption across both visible and UV regions. As shown in Fig. 6b, the absorption spectra exhibit high absorption coefficients, enhancing photon capture and carrier generation. The combination of favorable band-edge positioning, strong absorption capability, and narrow band gaps suggests potential suitability for photocatalytic applications. However, explicit assessment of charge separation efficiency and electron–hole recombination requires additional calculations beyond the scope of the present work. Therefore, Li_2_YCuCl_6_, Li_2_YCuBr_6_, and Li_2_YCuI_6_ emerge as promising candidates for solar-driven photocatalytic hydrogen production, with Li_2_YCuI_6_ predicted to excel in HER and Li_2_YCuCl_6_ offering the strongest driving force for OER. It is important to note that band-edge alignment and optical absorption alone cannot fully determine photocatalytic efficiency. Practical photocatalytic performance is also governed by factors such as surface activity, defect chemistry, charge-carrier mobility, carrier recombination, and reaction kinetics at the catalyst-water interface. Therefore, the present results should be regarded as a first-principles screening assessment of the photocatalytic potential of Li_2_YCuX_6_ (X = Cl, Br, I) rather than a definitive prediction of photocatalytic performance. Nevertheless, the favourable band-edge positions, visible-light absorption characteristics, and structural stability suggest that these materials represent promising candidates for further experimental and theoretical investigation.

## Conclusion

This study systematically investigated the structural, electronic, optical, mechanical, dynamical, and photocatalytic characteristics of Li_2_YCuX_6_ (X = Cl, Br, I) double perovskites as efficient photocatalysts for solar-driven water splitting. The compounds investigated exhibit thermodynamic stability, confirmed by negative formation energies and favorable Goldschmidt tolerance factors. Furthermore, phonon dispersion analysis reveals the absence of imaginary frequencies across the Brillouin zone, confirming the dynamical stability and lattice robustness of all compounds. All compounds exhibit thermal stability at 300 K without significant structural distortion, as confirmed by *ab initio* molecular dynamics (AIMD) simulations, where stable energy fluctuations over the simulation time indicate excellent thermal resilience under ambient conditions. Their semiconducting nature, together with suitable band gaps and strong UV-visible light absorption, suggests favorable characteristics for solar-energy conversion applications. The calculated band-edge alignments satisfy the thermodynamic requirements for both hydrogen evolution and oxygen evolution reactions, with Li_2_YCuI_6_ exhibiting the most negative conduction-band position among the investigated compounds. Overall, the combined structural, electronic, and optical characteristics indicate that Li_2_YCuX_6_ compounds possess promising attributes for photocatalytic water-splitting applications. However, practical photocatalytic performance is also influenced by factors such as surface activity, defect chemistry, charge-carrier transport, carrier recombination, and reaction kinetics, which were not explicitly investigated in the present work. Therefore, the current findings should be regarded as a first-principles screening assessment that identifies Li_2_YCuX_6_ double perovskites as promising candidates for further theoretical and experimental investigation.

## Conflicts of interest

All authors declare that they have no conflicts of interest.

## Data Availability

The data will be provided on request by the corresponding author.
